# 1,1′-Bis(3-methyl-3-phenyl­cyclo­but­yl)-2,2′-(aza­nedi­yl)diethanol

**DOI:** 10.1107/S1600536812010203

**Published:** 2012-03-14

**Authors:** Fatih Şen, Muharrem Dinçer, Alaaddin Çukurovalı, Ibrahim Yılmaz

**Affiliations:** a Kilis 7 Aralık University, Vocational High School of Health Services, Department of Opticianry, 79000 Kilis, Turkey; b Ondokuz Mayıs University, Arts and Sciences Faculty, Department of Physics, 55139 Samsun, Turkey; c Fırat University, Sciences Faculty, Department of Chemistry, 23119 Elazığ, Turkey; d Karamanoğlu Mehmetbey University, Faculty of Science, Department of Chemistry, 70200 Karaman, Turkey

## Abstract

The title mol­ecule, C_26_H_35_NO_2_, contains two cyclo­butane rings that adopt butterfly conformations and are linked by a –CH(OH)CH_2_NHCH_2_CH(OH)– bridge. In the crystal, N—H⋯O, O—H⋯N and O—H⋯O hydrogen bonds together with C–H⋯π inter­actions link the molecules.

## Related literature

For applications of related compounds, see: Dehmlow & Schmidt (1990 ▶); Coghi *et al.* (1976 ▶). For the preparation, see: Zalipsky *et al.* (1983 ▶). For puckering of the cyclo­butane ring, see: Swenson *et al.* (1997 ▶); Allen (1984 ▶).
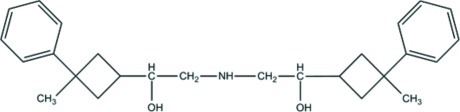


## Experimental

### 

#### Crystal data

C_26_H_35_NO_2_
*M* *_r_* = 393.55
Monoclinic, 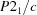

*a* = 6.2156 (4) Å
*b* = 33.2505 (15) Å
*c* = 12.1792 (8) Å
β = 110.656 (5)°
*V* = 2355.3 (2) Å^3^
*Z* = 4
Mo *K*α radiation
μ = 0.07 mm^−1^
*T* = 296 K
0.63 × 0.34 × 0.09 mm

#### Data collection

Stoe IPDS 2 diffractometer
Absorption correction: integration (*X-RED32*; Stoe & Cie, 2002 ▶) *T*_min_ = 0.967, *T*_max_ = 0.994
26921 measured reflections
4737 independent reflections
1740 reflections with *I* > 2σ(*I*)
*R*_int_ = 0.105

#### Refinement

*R*[*F* ^2^ > 2σ(*F* ^2^)] = 0.068
*wR*(*F* ^2^) = 0.173
*S* = 0.95
4737 reflections
271 parameters
2 restraints
H atoms treated by a mixture of independent and constrained refinement
Δρ_max_ = 0.29 e Å^−3^
Δρ_min_ = −0.13 e Å^−3^

### 

Data collection: *X-AREA* (Stoe & Cie, 2002 ▶); cell refinement: *X-AREA*; data reduction: *X-RED32* (Stoe & Cie, 2002 ▶); program(s) used to solve structure: *SHELXS97* (Sheldrick, 2008 ▶); program(s) used to refine structure: *SHELXL97* (Sheldrick, 2008 ▶); molecular graphics: *ORTEP-3 for Windows* (Farrugia, 1997 ▶); software used to prepare material for publication: *WinGX* (Farrugia, 1999 ▶) and *PLATON* (Spek, 2009 ▶).

## Supplementary Material

Crystal structure: contains datablock(s) global, I. DOI: 10.1107/S1600536812010203/sj5204sup1.cif

Structure factors: contains datablock(s) I. DOI: 10.1107/S1600536812010203/sj5204Isup2.hkl

Supplementary material file. DOI: 10.1107/S1600536812010203/sj5204Isup3.cml

Additional supplementary materials:  crystallographic information; 3D view; checkCIF report

## Figures and Tables

**Table 1:** Hydrogen-bond geometry (Å, °) *Cg*1 is the centroid of the C1–C6 ring.

*D*—H⋯*A*	*D*—H	H⋯*A*	*D*⋯*A*	*D*—H⋯*A*
N1—H1*N*⋯O2^i^	0.87 (3)	2.38 (3)	3.157 (4)	149 (3)
O1—H1*O*⋯N1^ii^	0.97 (2)	1.81 (3)	2.768 (4)	170 (3)
O2—H2*O*⋯O1)^i^	0.94 (3)	1.86 (3)	2.681 (4)	146 (3)
C24—H24⋯*Cg*1^iii^	0.93	3.86 (1)	2.76	156

Symmetry codes: (i) 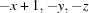; (ii) 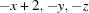; (iii) 
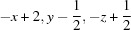
.

**Fig. 1.**
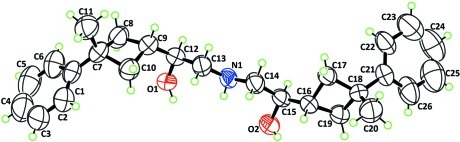
The structure of (I), showing the atom numbering scheme. Displacement ellipsoids are drawn at the 30% probability level.

**Fig. 2.**
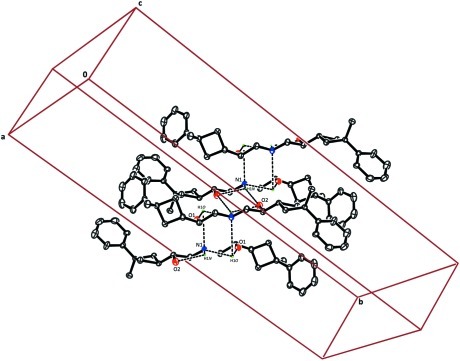
Part of the crystal structure of the title compound, showing the N—H···O and O—H···N interactions. For clarity, only H atoms involved in hydrogen bonding have been included. For symmetry codes, see Table 1.

**Fig. 3.**
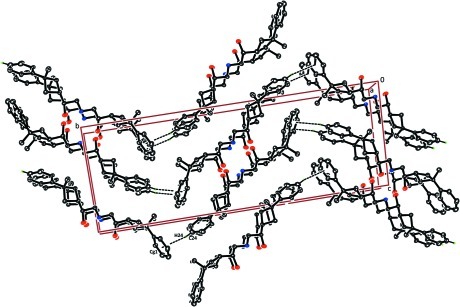
Part of the crystal structure of the title compound, showing the C—H···π interactions.For clarity, only H atoms involved in hydrogen bonding have been included.For symmetry codes, see table 1.

## supplementary crystallographic information

### Comment

It is well known that 3-substituted cyclobutane carboxylic acid derivatives exhibit anti-inflammatory and anti-depressant activity (Dehmlow & Schmidt, 1990), and also liquid crystal properties (Coghi, *et al.*, 1976).

The structure of (I) (Fig. 1) contains two cyclobutane rings (C7—C10),(C16—C19) each with methyl and phenyl substituents in the 3-position. The four-membered rings are linked by a C12,C13,N1,C14,C15 bridge. The best fit meanplanes through the (C7—C10) and (C16—C19) atoms of the cyclobutane rings subtend dihedral angles of 36.69 (24)°, 41.91 (21)° with the planes of the (C1—C6) and (C21—C26) phenyl rings respectively.

Values for the puckering of the cyclobutane has been reported as 23.5-24.3° (Swenson *et al.*. 1997, Allen, 1984). In this molecule the C7—C8—C9 plane forms a dihedral angle of 25.83 (43)° with the C9—C10—C7 plane and the angle between the C16—C17—C18 and C18—C19—C16 planes is 26.74 (36)°.

In the crystal structure N—H···O, O—H···N and C—H···π interactions stabilize the packing, Table 2, and link the molecules into infinite chains, Fig.2, Fig. 3.

### Experimental

The compound was synthesised using a literature method (Zalipsky *et al.*, 1983) with some modification. Colourless plate-like crystals suitable for X-ray analysis were obtained by crystallization from ethanol. Overall yield: 71%. *M*.p.: 445 K (EtOH).

### Refinement

H atoms were positioned geometrically and treated using a riding model, with bond lengths 0.96, 0.97, 0.98 and 0.93 Å for CH_3_, CH_2_, CH and CH (aromatic), respectively. H atoms bound to the N and O atoms were located in difference maps and refined with DFIX restraints N—H = 0.87 (3) Å and O—H = 0.82 (2) Å. The displacement parameters of the H atoms bound to C were constrained with U_iso_(H) = 1.2 (aromatic, methylene or methine C) or 1.5*U*_eq_ (methyl C).

### Crystal data

**Table:**

C_26_H_35_NO_2_	*F*(000) = 856
*M**_r_* = 393.55	*D*_x_ = 1.110 Mg m^−^^3^
Monoclinic, *P*2_1_/*c*	Mo *K*α radiation, λ = 0.71073 Å
Hall symbol: -P 2ybc	Cell parameters from 18482 reflections
*a* = 6.2156 (4) Å	θ = 1.2–26.7°
*b* = 33.2505 (15) Å	µ = 0.07 mm^−^^1^
*c* = 12.1792 (8) Å	*T* = 296 K
β = 110.656 (5)°	Plate, colourless
*V* = 2355.3 (2) Å^3^	0.63 × 0.34 × 0.09 mm
*Z* = 4	

### Data collection

**Table:**

Stoe IPDS 2 diffractometer	4737 independent reflections
Radiation source: fine-focus sealed tube	1740 reflections with *I* > 2σ(*I*)
Graphite monochromator	*R*_int_ = 0.105
Detector resolution: 6.67 pixels mm^-1^	θ_max_ = 26.3°, θ_min_ = 1.2°
rotation method scans	*h* = −7→7
Absorption correction: integration (*X-RED32*; Stoe & Cie, 2002)	*k* = −41→41
*T*_min_ = 0.967, *T*_max_ = 0.994	*l* = −15→15
26921 measured reflections	

### Refinement

**Table:**

Refinement on *F*^2^	Primary atom site location: structure-invariant direct methods
Least-squares matrix: full	Secondary atom site location: difference Fourier map
*R*[*F*^2^ > 2σ(*F*^2^)] = 0.068	Hydrogen site location: inferred from neighbouring sites
*wR*(*F*^2^) = 0.173	H atoms treated by a mixture of independent and constrained refinement
*S* = 0.95	*w* = 1/[σ^2^(*F*_o_^2^) + (0.0627*P*)^2^] where *P* = (*F*_o_^2^ + 2*F*_c_^2^)/3
4737 reflections	(Δ/σ)_max_ < 0.001
271 parameters	Δρ_max_ = 0.29 e Å^−^^3^
2 restraints	Δρ_min_ = −0.13 e Å^−^^3^

### Special details

**Table:**

Experimental. IR (KBr, ν cm-1): 3416 (–OH), 3288 (–NH–), 3089–3024 (aromatics), 2960–2858 (aliphatics), 1497 (C—N), 1113 (C—O), ^1^H NMR (CDCl_3_, TMS, δ p.p.m.): 1.46 (s, 6H,–CH_3_), 2.08 (d,*j* = 8.8 Hz, 4H, CH_2_– in cyclobutane ring), 2.21 (d, *j* = 8.4 Hz, 4H, –CH_2_– in cyclobutane ring), 2.32–2.42 (m, 4H, CH2-), 2.59 (dd, *j*=12.0 Hz, 2H, >CH–), 3.15 (brs, 3H, –OH plus –NH–),3.50 (quint, *j_1_*=7.4 Hz, *j_2_*=2.4 Hz, 2H, >CH–, in cyclobutane), 7.13–7.20 (m, 6H, aromatics), 7.29–7.33 (m, 4H, aromatics). ^13^C NMR (CDCl_3_, TMS, δ p.p.m.): 152.47, 128.20, 125.26, 124.66, 74.27, 53.20, 38.77, 36.80, 36.14, 33.15, 30.70.
Geometry. All e.s.d.'s (except the e.s.d. in the dihedral angle between two l.s. planes) are estimated using the full covariance matrix. The cell e.s.d.'s are taken into account individually in the estimation of e.s.d.'s in distances, angles and torsion angles; correlations between e.s.d.'s in cell parameters are only used when they are defined by crystal symmetry. An approximate (isotropic) treatment of cell e.s.d.'s is used for estimating e.s.d.'s involving l.s. planes.
Refinement. Refinement of *F*^2^ against ALL reflections. The weighted *R*-factor *wR* and goodness of fit *S* are based on *F*^2^, conventional *R*-factors *R* are based on *F*, with *F* set to zero for negative *F*^2^. The threshold expression of *F*^2^ > σ(*F*^2^) is used only for calculating *R*-factors(gt) *etc*. and is not relevant to the choice of reflections for refinement. *R*-factors based on *F*^2^ are statistically about twice as large as those based on *F*, and *R*- factors based on ALL data will be even larger.

### Fractional atomic coordinates and isotropic or equivalent isotropic displacement parameters (Å^2^)

**Table:**

	*x*	*y*	*z*	*U*_iso_*/*U*_eq_	
C1	0.8359 (8)	0.20105 (13)	−0.1639 (4)	0.1058 (13)	
H1	0.7569	0.2015	−0.1117	0.127*	
C2	0.7425 (10)	0.21975 (15)	−0.2715 (6)	0.140 (2)	
H2	0.6004	0.2324	−0.2916	0.168*	
C3	0.8559 (17)	0.2198 (2)	−0.3480 (6)	0.165 (3)	
H3	0.7941	0.2330	−0.4196	0.198*	
C4	1.0599 (15)	0.2005 (2)	−0.3198 (6)	0.154 (2)	
H4	1.1362	0.1997	−0.3731	0.184*	
C5	1.1544 (9)	0.18199 (13)	−0.2128 (4)	0.1127 (15)	
H5	1.2963	0.1693	−0.1942	0.135*	
C6	1.0466 (8)	0.18161 (11)	−0.1327 (4)	0.0830 (11)	
C7	1.1539 (6)	0.16222 (10)	−0.0146 (3)	0.0773 (10)	
C8	1.2839 (6)	0.12244 (11)	−0.0088 (4)	0.0976 (12)	
H8A	1.2404	0.1085	−0.0833	0.117*	
H8B	1.4496	0.1251	0.0261	0.117*	
C9	1.1733 (6)	0.10506 (11)	0.0749 (3)	0.0848 (11)	
H9	1.2729	0.1100	0.1563	0.102*	
C10	0.9910 (6)	0.13874 (10)	0.0332 (3)	0.0834 (10)	
H10A	0.8492	0.1302	−0.0272	0.100*	
H10B	0.9604	0.1523	0.0966	0.100*	
C11	1.3001 (7)	0.19339 (12)	0.0741 (4)	0.1111 (14)	
H11A	1.3692	0.1809	0.1495	0.167*	
H11B	1.2038	0.2152	0.0803	0.167*	
H11C	1.4183	0.2035	0.0479	0.167*	
C12	1.0931 (6)	0.06167 (11)	0.0605 (3)	0.0784 (10)	
H12	1.2276	0.0441	0.0781	0.094*	
C13	0.9725 (6)	0.05249 (11)	0.1454 (3)	0.0858 (11)	
H13A	1.0804	0.0563	0.2246	0.103*	
H13B	0.8486	0.0717	0.1329	0.103*	
C14	0.7920 (6)	0.00309 (10)	0.2295 (3)	0.0822 (10)	
H14A	0.6836	0.0239	0.2311	0.099*	
H14B	0.9194	0.0039	0.3038	0.099*	
C15	0.6758 (6)	−0.03736 (10)	0.2161 (3)	0.0742 (10)	
H15	0.7914	−0.0581	0.2221	0.089*	
C16	0.5795 (5)	−0.04484 (10)	0.3104 (3)	0.0694 (9)	
H16	0.4640	−0.0244	0.3076	0.083*	
C17	0.7514 (5)	−0.04950 (9)	0.4367 (3)	0.0697 (9)	
H17A	0.9025	−0.0585	0.4407	0.084*	
H17B	0.7628	−0.0258	0.4849	0.084*	
C18	0.6036 (5)	−0.08305 (9)	0.4600 (3)	0.0644 (9)	
C19	0.4902 (6)	−0.08705 (11)	0.3251 (3)	0.0842 (11)	
H19A	0.5548	−0.1084	0.2922	0.101*	
H19B	0.3239	−0.0889	0.2977	0.101*	
C20	0.4371 (6)	−0.06601 (13)	0.5146 (4)	0.1093 (14)	
H20A	0.3630	−0.0426	0.4716	0.164*	
H20B	0.5200	−0.0588	0.5947	0.164*	
H20C	0.3233	−0.0859	0.5117	0.164*	
C21	0.7205 (7)	−0.11958 (11)	0.5263 (3)	0.0751 (10)	
C22	0.9511 (7)	−0.11942 (13)	0.5945 (3)	0.0999 (13)	
H22	1.0387	−0.0963	0.6001	0.120*	
C23	1.0512 (11)	−0.1540 (2)	0.6546 (5)	0.157 (3)	
H23	1.2071	−0.1538	0.6996	0.189*	
C24	0.9279 (19)	−0.1881 (2)	0.6496 (6)	0.183 (4)	
H24	0.9979	−0.2111	0.6898	0.220*	
C25	0.6985 (17)	−0.18775 (16)	0.5840 (6)	0.176 (3)	
H25	0.6107	−0.2106	0.5813	0.211*	
C26	0.5959 (9)	−0.15427 (13)	0.5223 (4)	0.1241 (16)	
H26	0.4402	−0.1549	0.4771	0.149*	
O1	0.9385 (4)	0.05398 (7)	−0.0596 (2)	0.0841 (7)	
O2	0.5073 (4)	−0.03918 (9)	0.1009 (2)	0.1019 (9)	
N1	0.8774 (5)	0.01166 (9)	0.1352 (2)	0.0789 (9)	
H1N	0.761 (6)	0.0091 (10)	0.070 (3)	0.095*	
H1O	0.986 (6)	0.0304 (9)	−0.092 (3)	0.118*	
H2O	0.371 (5)	−0.0403 (12)	0.117 (3)	0.118*	

### Atomic displacement parameters (Å^2^)

**Table:**

	*U*^11^	*U*^22^	*U*^33^	*U*^12^	*U*^13^	*U*^23^
C1	0.107 (3)	0.084 (3)	0.123 (4)	−0.010 (3)	0.037 (3)	0.009 (3)
C2	0.137 (5)	0.099 (4)	0.146 (5)	−0.023 (3)	0.001 (4)	0.037 (4)
C3	0.227 (9)	0.115 (5)	0.110 (5)	−0.072 (5)	0.006 (6)	0.025 (4)
C4	0.230 (8)	0.134 (5)	0.105 (5)	−0.055 (5)	0.069 (5)	−0.001 (4)
C5	0.148 (4)	0.100 (3)	0.105 (4)	−0.019 (3)	0.064 (4)	0.001 (3)
C6	0.099 (3)	0.063 (2)	0.092 (3)	−0.022 (2)	0.041 (3)	−0.010 (2)
C7	0.079 (2)	0.067 (2)	0.090 (3)	−0.019 (2)	0.035 (2)	−0.009 (2)
C8	0.083 (2)	0.082 (3)	0.139 (4)	−0.001 (2)	0.053 (3)	0.010 (2)
C9	0.075 (2)	0.081 (3)	0.090 (3)	−0.013 (2)	0.019 (2)	0.007 (2)
C10	0.084 (2)	0.081 (2)	0.091 (3)	0.000 (2)	0.038 (2)	0.000 (2)
C11	0.117 (3)	0.099 (3)	0.105 (3)	−0.027 (3)	0.026 (3)	−0.011 (3)
C12	0.069 (2)	0.085 (3)	0.071 (2)	−0.0025 (19)	0.012 (2)	0.011 (2)
C13	0.092 (2)	0.079 (3)	0.078 (2)	−0.010 (2)	0.020 (2)	0.011 (2)
C14	0.093 (3)	0.080 (3)	0.065 (2)	−0.006 (2)	0.018 (2)	0.0078 (19)
C15	0.073 (2)	0.081 (3)	0.055 (2)	−0.0028 (19)	0.0054 (19)	0.0093 (18)
C16	0.0628 (19)	0.069 (2)	0.071 (2)	0.0057 (17)	0.0173 (18)	0.0129 (18)
C17	0.071 (2)	0.072 (2)	0.061 (2)	−0.0004 (17)	0.0162 (18)	0.0022 (17)
C18	0.0564 (19)	0.070 (2)	0.068 (2)	0.0012 (17)	0.0227 (17)	0.0045 (18)
C19	0.075 (2)	0.090 (3)	0.073 (2)	−0.0131 (19)	0.0066 (19)	0.007 (2)
C20	0.093 (3)	0.119 (3)	0.131 (4)	0.022 (2)	0.060 (3)	0.025 (3)
C21	0.096 (3)	0.069 (2)	0.063 (2)	0.011 (2)	0.031 (2)	0.0043 (18)
C22	0.097 (3)	0.122 (3)	0.084 (3)	0.038 (3)	0.036 (2)	0.034 (3)
C23	0.173 (5)	0.194 (7)	0.118 (4)	0.101 (6)	0.068 (4)	0.074 (5)
C24	0.317 (12)	0.146 (6)	0.104 (5)	0.133 (8)	0.096 (6)	0.065 (5)
C25	0.327 (10)	0.069 (4)	0.123 (5)	0.001 (5)	0.069 (6)	0.023 (3)
C26	0.172 (4)	0.077 (3)	0.107 (3)	−0.017 (3)	0.030 (3)	0.012 (3)
O1	0.0786 (15)	0.0864 (17)	0.0791 (17)	0.0006 (13)	0.0177 (13)	0.0055 (14)
O2	0.0920 (18)	0.128 (2)	0.0659 (16)	−0.0200 (17)	0.0035 (15)	0.0200 (15)
N1	0.090 (2)	0.084 (2)	0.0557 (18)	−0.0114 (18)	0.0164 (15)	0.0083 (16)

### Geometric parameters (Å, º)

**Table:**

C1—C2	1.381 (6)	C14—H14A	0.9700
C1—C6	1.388 (5)	C14—H14B	0.9700
C1—H1	0.9300	C15—O2	1.426 (3)
C2—C3	1.352 (9)	C15—C16	1.491 (4)
C2—H2	0.9300	C15—H15	0.9800
C3—C4	1.353 (8)	C16—C17	1.540 (4)
C3—H3	0.9300	C16—C19	1.543 (4)
C4—C5	1.372 (7)	C16—H16	0.9800
C4—H4	0.9300	C17—C18	1.534 (4)
C5—C6	1.365 (5)	C17—H17A	0.9700
C5—H5	0.9300	C17—H17B	0.9700
C6—C7	1.500 (5)	C18—C21	1.497 (4)
C7—C8	1.538 (5)	C18—C20	1.523 (4)
C7—C11	1.541 (4)	C18—C19	1.548 (4)
C7—C10	1.546 (4)	C19—H19A	0.9700
C8—C9	1.529 (5)	C19—H19B	0.9700
C8—H8A	0.9700	C20—H20A	0.9600
C8—H8B	0.9700	C20—H20B	0.9600
C9—C12	1.516 (5)	C20—H20C	0.9600
C9—C10	1.546 (4)	C21—C22	1.380 (4)
C9—H9	0.9800	C21—C26	1.380 (5)
C10—H10A	0.9700	C22—C23	1.387 (6)
C10—H10B	0.9700	C22—H22	0.9300
C11—H11A	0.9600	C23—C24	1.358 (9)
C11—H11B	0.9600	C23—H23	0.9300
C11—H11C	0.9600	C24—C25	1.367 (9)
C12—O1	1.462 (4)	C24—H24	0.9300
C12—C13	1.506 (5)	C25—C26	1.368 (7)
C12—H12	0.9800	C25—H25	0.9300
C13—N1	1.469 (4)	C26—H26	0.9300
C13—H13A	0.9700	O1—H1O	0.97 (2)
C13—H13B	0.9700	O2—H2O	0.93 (2)
C14—N1	1.453 (4)	N1—H1N	0.87 (3)
C14—C15	1.508 (4)		
			
C2—C1—C6	120.6 (5)	N1—C14—H14B	109.1
C2—C1—H1	119.7	C15—C14—H14B	109.1
C6—C1—H1	119.7	H14A—C14—H14B	107.8
C3—C2—C1	120.6 (7)	O2—C15—C16	113.3 (3)
C3—C2—H2	119.7	O2—C15—C14	107.6 (3)
C1—C2—H2	119.7	C16—C15—C14	111.9 (3)
C2—C3—C4	119.6 (8)	O2—C15—H15	108.0
C2—C3—H3	120.2	C16—C15—H15	108.0
C4—C3—H3	120.2	C14—C15—H15	108.0
C3—C4—C5	120.2 (7)	C15—C16—C17	117.4 (3)
C3—C4—H4	119.9	C15—C16—C19	120.0 (3)
C5—C4—H4	119.9	C17—C16—C19	86.8 (2)
C6—C5—C4	122.0 (5)	C15—C16—H16	110.2
C6—C5—H5	119.0	C17—C16—H16	110.2
C4—C5—H5	119.0	C19—C16—H16	110.2
C5—C6—C1	117.0 (4)	C18—C17—C16	90.5 (2)
C5—C6—C7	121.7 (4)	C18—C17—H17A	113.6
C1—C6—C7	121.3 (4)	C16—C17—H17A	113.6
C6—C7—C8	117.5 (3)	C18—C17—H17B	113.6
C6—C7—C11	109.6 (3)	C16—C17—H17B	113.6
C8—C7—C11	112.1 (3)	H17A—C17—H17B	110.8
C6—C7—C10	116.7 (3)	C21—C18—C20	110.0 (3)
C8—C7—C10	87.2 (3)	C21—C18—C17	118.8 (3)
C11—C7—C10	112.2 (3)	C20—C18—C17	110.7 (3)
C9—C8—C7	90.2 (3)	C21—C18—C19	117.1 (3)
C9—C8—H8A	113.6	C20—C18—C19	111.7 (3)
C7—C8—H8A	113.6	C17—C18—C19	86.8 (2)
C9—C8—H8B	113.6	C16—C19—C18	89.9 (2)
C7—C8—H8B	113.6	C16—C19—H19A	113.7
H8A—C8—H8B	110.9	C18—C19—H19A	113.7
C12—C9—C8	119.3 (3)	C16—C19—H19B	113.7
C12—C9—C10	118.6 (3)	C18—C19—H19B	113.7
C8—C9—C10	87.5 (3)	H19A—C19—H19B	110.9
C12—C9—H9	109.9	C18—C20—H20A	109.5
C8—C9—H9	109.9	C18—C20—H20B	109.5
C10—C9—H9	109.9	H20A—C20—H20B	109.5
C7—C10—C9	89.3 (3)	C18—C20—H20C	109.5
C7—C10—H10A	113.8	H20A—C20—H20C	109.5
C9—C10—H10A	113.8	H20B—C20—H20C	109.5
C7—C10—H10B	113.8	C22—C21—C26	118.4 (4)
C9—C10—H10B	113.8	C22—C21—C18	121.7 (3)
H10A—C10—H10B	111.0	C26—C21—C18	120.0 (4)
C7—C11—H11A	109.5	C21—C22—C23	119.5 (5)
C7—C11—H11B	109.5	C21—C22—H22	120.3
H11A—C11—H11B	109.5	C23—C22—H22	120.3
C7—C11—H11C	109.5	C24—C23—C22	121.9 (7)
H11A—C11—H11C	109.5	C24—C23—H23	119.1
H11B—C11—H11C	109.5	C22—C23—H23	119.1
O1—C12—C13	109.9 (3)	C23—C24—C25	118.3 (6)
O1—C12—C9	110.8 (3)	C23—C24—H24	120.8
C13—C12—C9	109.7 (3)	C25—C24—H24	120.8
O1—C12—H12	108.8	C24—C25—C26	121.1 (7)
C13—C12—H12	108.8	C24—C25—H25	119.5
C9—C12—H12	108.8	C26—C25—H25	119.5
N1—C13—C12	114.4 (3)	C25—C26—C21	120.9 (5)
N1—C13—H13A	108.7	C25—C26—H26	119.5
C12—C13—H13A	108.7	C21—C26—H26	119.5
N1—C13—H13B	108.7	C12—O1—H1O	111 (2)
C12—C13—H13B	108.7	C15—O2—H2O	102 (2)
H13A—C13—H13B	107.6	C14—N1—C13	111.3 (3)
N1—C14—C15	112.6 (3)	C14—N1—H1N	106 (2)
N1—C14—H14A	109.1	C13—N1—H1N	110 (2)
C15—C14—H14A	109.1		
			
C6—C1—C2—C3	−0.7 (7)	N1—C14—C15—C16	−177.3 (3)
C1—C2—C3—C4	1.7 (9)	O2—C15—C16—C17	171.5 (3)
C2—C3—C4—C5	−2.0 (10)	C14—C15—C16—C17	−66.6 (4)
C3—C4—C5—C6	1.3 (8)	O2—C15—C16—C19	68.5 (4)
C4—C5—C6—C1	−0.3 (6)	C14—C15—C16—C19	−169.7 (3)
C4—C5—C6—C7	−178.4 (4)	C15—C16—C17—C18	−140.9 (3)
C2—C1—C6—C5	0.0 (6)	C19—C16—C17—C18	−18.6 (3)
C2—C1—C6—C7	178.1 (4)	C16—C17—C18—C21	137.9 (3)
C5—C6—C7—C8	−39.3 (5)	C16—C17—C18—C20	−93.5 (3)
C1—C6—C7—C8	142.6 (3)	C16—C17—C18—C19	18.6 (2)
C5—C6—C7—C11	90.2 (4)	C15—C16—C19—C18	138.4 (3)
C1—C6—C7—C11	−87.9 (4)	C17—C16—C19—C18	18.5 (2)
C5—C6—C7—C10	−140.9 (4)	C21—C18—C19—C16	−139.4 (3)
C1—C6—C7—C10	41.1 (5)	C20—C18—C19—C16	92.5 (3)
C6—C7—C8—C9	−136.8 (3)	C17—C18—C19—C16	−18.5 (2)
C11—C7—C8—C9	94.8 (3)	C20—C18—C21—C22	−110.3 (4)
C10—C7—C8—C9	−18.1 (3)	C17—C18—C21—C22	18.7 (5)
C7—C8—C9—C12	139.7 (3)	C19—C18—C21—C22	120.7 (3)
C7—C8—C9—C10	18.1 (3)	C20—C18—C21—C26	68.1 (4)
C6—C7—C10—C9	137.4 (3)	C17—C18—C21—C26	−162.9 (4)
C8—C7—C10—C9	17.9 (3)	C19—C18—C21—C26	−60.8 (4)
C11—C7—C10—C9	−94.9 (3)	C26—C21—C22—C23	1.3 (6)
C12—C9—C10—C7	−140.3 (4)	C18—C21—C22—C23	179.7 (4)
C8—C9—C10—C7	−18.0 (3)	C21—C22—C23—C24	−0.8 (8)
C8—C9—C12—O1	−53.6 (4)	C22—C23—C24—C25	−0.6 (11)
C10—C9—C12—O1	50.8 (5)	C23—C24—C25—C26	1.7 (11)
C8—C9—C12—C13	−175.1 (3)	C24—C25—C26—C21	−1.2 (9)
C10—C9—C12—C13	−70.7 (4)	C22—C21—C26—C25	−0.3 (7)
O1—C12—C13—N1	55.1 (4)	C18—C21—C26—C25	−178.8 (4)
C9—C12—C13—N1	177.2 (3)	C15—C14—N1—C13	175.4 (3)
N1—C14—C15—O2	−52.2 (4)	C12—C13—N1—C14	172.2 (3)

### Hydrogen-bond geometry (Å, º)

Cg1 is the centroid of the C1–C6 ring.

**Table:**

*D*—H···*A*	*D*—H	H···*A*	*D*···*A*	*D*—H···*A*
N1—H1*N*···O2^i^	0.87 (3)	2.38 (3)	3.157 (4)	149 (3)
O1—H1*O*···N1^ii^	0.97 (2)	1.81 (3)	2.768 (4)	170 (3)
O2—H2*O*···O1)^i^	0.94 (3)	1.86 (3)	2.681 (4)	146 (3)
C24—H24···*Cg*1^iii^	0.93	3.86 (1)	2.76	156

Symmetry codes: (i) −*x*+1, −*y*, −*z*; (ii) −*x*+2, −*y*, −*z*; (iii) −*x*+2, *y*−1/2, −*z*+1/2.
